# Potential Therapeutic Roles for Inhibition of the PI3K/Akt/mTOR Pathway in the Pathophysiology of Diabetic Retinopathy

**DOI:** 10.1155/2011/589813

**Published:** 2011-10-30

**Authors:** Jorge L. Jacot, David Sherris

**Affiliations:** ^1^Department of Pathology and Anatomy, Eastern Virginia Medical School, Norfolk, VA, USA; ^2^Angioceutics International, L.L.C., 1141 Kings Way Drive, Virginia Beach, VA 23455, USA; ^3^Paloma Pharmaceuticals, Inc., 37 Neillian Crescent, Jamaica Plain, MA 02130, USA

## Abstract

Novel therapeutics such as inhibitors of PI3K/Akt/mTOR pathway presents a unique opportunity for the management of diabetic retinopathy (DR). Second generation mTOR inhibitors have the prospect to be efficacious in managing various stages of disease progression in DR. During early stages, the mTOR inhibitors suppress HIF-1**α**, VEGF, leakage, and breakdown of the blood-retinal barrier. These mTOR inhibitors impart a pronounced inhibitory effect on inflammation, an early component with diverse ramifications influencing the progression of DR. These inhibitors suppress IKK and NF-**κ**B along with downstream inflammatory cytokines, chemokines, and adhesion molecules. In proliferative DR, mTOR inhibitors suppress several growth factors that play pivotal roles in the induction of pathological angiogenesis. Lead mTOR inhibitors in clinical trials for ocular indications present an attractive treatment option for chronic use in DR with favorable safety profile and sustained ocular pharmacokinetics following single dose. Thereby, reducing dosing frequency and risk associated with chronic drug administration.

## 1. Introduction

Blindness as a consequence of diabetic retinopathy from long-standing or poorly controlled diabetes causes profound adverse psychological effects to the diabetic patient. Diabetic retinopathy has a significant economic impact on society in terms of healthcare resources that are required and the potential of loss in the workforce. The number of people at risk of blindness from diabetic retinopathy in the United States alone continues to rise, and diabetic retinopathy is the leading cause of blindness in the industrialized world covering a wide age range in adults [1]. Diabetic retinopathy affects 75% of all diabetics after 15 years of the disease and up to 97.5% after 15 years of the disease when diagnosis is made prior to 30 years of age [2]. One in five patients will progress to develop proliferative retinopathy after 25 years of known diabetes [2] Predictions for the prevalence of diabetic retinopathy in the USA over the next 39 years for those older than 40 years are 16 million and for those over 65 years are 9.9 million [3]. Moreover, by the year 2050, those afflicted with a sight-threatening stage of proliferative diabetic retinopathy are projected to be 3.4 million for those over 40 years of age and 1.9 million for those 65 years of age or older [3]. Tight control of presumed key risk factors now appears to be insufficient in minimizing the prevalence of sight-threatening proliferative retinopathy [4]. In addition to the established risk factors, genomic linkage analysis suggests evidence for a genetic predisposition to develop diabetic retinopathy [5]. It is clear that breakthrough treatment options and targeted intervention approaches are needed to make inroads into the treatment of this devastating disease that threatens a growing number of diabetics.

## 2. Current Pharmacological Options to Combat Angiogenesis in Diabetic Retinopathy

Anti-VEGF-A therapeutics has become a dominant approach for the management of ocular neovascular diseases [6, 7]. Ongoing clinical trials for diabetic retinopathy predominantly focus on a mechanism of action mediated via VEGF-A antagonism. Of the 103 currently open NIH-sponsored clinical trials involving diabetic retinopathy, the majorities are aimed at treatment of diabetic macular edema and proliferative diabetic retinopathy using Lucentis (ranibizumab), Avastin (Bevacizumab), and to a lesser extent Macugen (pegaptanib) either as sole agents, in combination with other pharmacological agents, or in combination with laser photocoagulation therapy (Table 1). 

Within the past seven years, two drugs targeting VEGF were approved for combating ocular neovascularization. Both these drugs, Macugen (pegaptanib) and Lucentis (ranibizumab) were approved for exudative age-related macular degeneration. More recently, Lucentis has received approval for use in patients suffering visual impairment due to macular edema secondary to central and branch retinal vein occlusion [8, 9]. The anti-VEGF monoclonal antibody drug Avastin is currently used off-label for wet macular degeneration. 

The success of anti-VEGF treatments has produced an unprecedented understanding of the factors and pathogenic mechanisms operant in several retinal neovascular diseases and has demonstrated that therapeutic agents considered initially only in the realm of anticancer agents have demonstrated efficacy in combating ocular neovascularization. Could a similar story be on the horizon for mTOR inhibitors for which the principal indication has also been in the treatment of cancers?

Other antiangiogenic approaches for ocular angiogenic diseases involve growth factors (GH, IGF-1), steroid compounds, or kinase inhibitors (PKC, Src). No mTOR inhibitors which target the mammalian target of rapamycin are currently being clinically evaluated for their efficacy in nonproliferative or proliferative stages of diabetic retinopathy. Only two mTOR compounds, Sirolimus (MacuSight, Union City, Calif, USA) and Palomid 529 (Paloma Pharmaceuticals, Inc. Jamaica Plain, Mass, USA) are currently being evaluated in NIH-sponsored trials for ocular indications. Sirolimus is being evaluated to treat diabetic macular edema which is a frequent manifestation of diabetic retinopathy, for ARMD, and for uveitis. Palomid 529 is being evaluated for ARMD [6, 10]. The current review presents the rational basis for the utility of mTOR inhibitors in addressing some of the known pathophysiological events that occur during the early development and late stage progression of diabetic retinopathy and how the mTOR inhibitors could be a potentially efficacious option in the management of diabetic retinopathy.

## 3. Involvement of the Phosphatidylinositol-3-kinase/AKt/Mammalian Target of Rapamycin (PI3K/Akt/mTOR) Pathway in Hyperglycemic Vasculopathy

An active PI3K/Akt pathway has been linked to glucose dysmetabolism in retinal tissue. The direct effect of high glucose on retinal endothelial cells imparts a promigratory phenotype with increased fibronectin and alpha (v) beta-(3) integrin expressions which appears to be concomitant with the activation of PI3K/Akt pathway [11]. Elevated glucose levels cause decreased uptake of 2-deoxyglucose as a consequence of downregulated expression of GLUT-1 transporter. The dysmetabolism of glucose utilization and downregulation of GLUT-1 are mediated by the PI3K and Akt pathways since pharmacological inhibition of PI3K and Akt preserved GLUT-1 expression [12]. 

Experimental findings [13] suggest that Akt interaction with RhoB may subserve endothelial cell survival during vascular development and perhaps pathological angiogenesis leading to the microangiopathies characteristic of diabetic microvascular disease. It can be surmised that the inhibition of the PI3K/Akt/mTOR pathway that disrupt the Akt-RhoB interaction could promote endothelial cell death. Prevention of endothelial cell proliferation and enhancement of endothelial cell apoptosis could serve as a treatment modality to delay or prevent progression of vasculopathies observed in diabetic retinopathy since the phenotype of enhanced migration of endothelial cells is a requirement for neovascularization to occur.

The *in vitro* finding that the mRNA and protein expression of the anti-angiogenic factor PEDF is reduced by glucose as well as insulin raises interesting implications for the diabetic retina. These common physiological metabolites are elevated in type 2 diabetics, and it has been shown that these metabolites are dependent on the mTOR pathway for their destabilizing effect on PEDF [14]. Therefore, mTOR inhibition may stabilize PEDF mRNA along with protein expression levels and promote an anti-angiogenic milieu in the diabetic retina.

## 4. Significance of HIF-1*α*, VEGF, and mTOR Inhibition in Preproliferative Diabetic Retinopathy

The DCCT study highlighted a transient “early worsening” effect that occurs during acute management of diabetics with retinopathy [15]. In vitro studies investigating the underlying mechanistic factors responsible for the occurrence of early worsening suggest that the phenomenon appears to stem from a hypoxic retina as a consequence of compromised retinal hemodynamics in conjunction with low-glucose availability [16]. The hypoxia is exacerbated by an acute reduction of available glucose due to the “tight” glucose control. Intensive lowering of glucose by insulin could result in insufficient glucose to meet retinal metabolic requirements. Concomitantly, the acute intensive insulin treatment could induce HIF-*α* expression via PI3K-dependent pathway [17].

HIF-1*α* is a principal regulator of VEGF expression. The binding of HIF-1*α* to the VEGF hypoxia-responsive elements promoter evokes signaling via MAPK, PI3K, and JNK pathways with a resultant increase in VEGF expression. The Src kinase pathway leads to VEGF-mediated retinal vascular permeability and breakdown of blood-retinal barrier that may be observed in diabetes [18]. An increase in permeability of the endothelium in diabetes involves VEGF in conjunction with PKC activation. VEGF promotes the phosphorylation of the tight-junction complex protein occludin via a PKC-dependent pathway [19]. Further evidence for the central involvement of VEGF is the observation that VEGF immunoreactivity is correlated with vascular leakage of macromolecules in human diabetic retinas [20]. Additionally, chimeric antibodies that sequester VEGF bioavailability (“VEGF-trap”) reduce vascular leakage as demonstrated by reduction in extravasation of Evans blue dye in the retina [21, 22]. An increased VEGF level promotes an acute breakdown of the blood-retinal barrier that clinically manifests as retinal edema and exudates in diabetic patients. The breakdown of the blood-retinal barrier accounts for the clinical manifestations of “early worsening” effect in patients with minimal to moderate retinopathy. 

The mTOR inhibitors have the potential to suppress the occurrence and or severity of the transient “early worsening” effect by helping to avert breakdown of blood-retinal barrier by modulating HIF-1*α*-mediated downstream activation of growth factors, such as the transcriptional regulation of retinal VEGF. The timing of this intervention would precede the development of irreversible structural damage to the retinal microvasculature and could have a profound effect in curtailing future deleterious events and perhaps delay or prevent the progression of retinal microangiopathies.

## 5. Link between Inflammation, Oxidative Stress, PI3K/Akt/mTOR, and Progressive Diabetic Retinopathy

The natural history of diabetic retinopathy suggests that both chronic inflammatory and oxidative stress components appear to be operant in the development of progressive diabetic retinopathy [23]. Using gene-chip array technology applied to samples from streptozotocin-induced diabetic rats, the upregulation of several genes integral to inflammation, oxidative stress, apoptosis, TGF-*β*-signaling cascade, and additional genes related to vascular turnover of retinal blood vessels has been demonstrated [24]. In the diabetic retina, AGE modify proteins promote oxidative stress and increase inflammatory cytokines that alter vascular function [25]. Microglial-mediated release of TNF-*α* and IL-1*β* is a mechanism by which a pro-inflammatory environment exists in the diabetic retina and contributes to the development of experimental diabetic retinopathy. Lipid-soluble tetracycline class of antibiotics that attenuate TNF-*α* and NF-*κ*B suppress downstream inflammatory mediators and pro-apoptotic signals derived from activated retinal microglial cells [26].

An increasing body of evidence suggests that a localized inflammatory process that resides within the retina is integral to the early development of diabetic retinopathy. This inflammatory process results in a local increase of iNOS, NF-*κ*B, IL-1*β*, cytokines, caspases, COX-2, PGE2, the adhesion molecule intercellular adhesion molecule (ICAM-1), VEGF, and increased permeability and leukostasis within the retina [27]. 

The characteristic microangiopathy that develops in diabetic retinopathy is linked to localized inflammation. An early hemodynamic change observed in the diabetic retina of animal models and humans is an increase in leukostasis and increased expression of cell adhesion molecules such as ICAM-1 and P-selectin [28]. Mice deficient in TNF-alpha exhibit extensive reduction in leukocytosis in the retinal vessels suggesting that the pro-inflammatory cytokine contributes to the leukostasis triggered by platelet-activating factor, IL-1*β*, and VEGF [29]. Evidence that leukostasis in diabetic retinopathy is linked to oxidant stress and other downstream mediators comes from the observation that alpha-lipoic acid abrogates increases in leukocyte adhesion while other mechanisms, linked to PKC pathways, are responsible for hemodynamic alterations that occur concomitantly with leukostasis [30].

In a diabetic nonhuman primate model, the elevated circulating numbers of polymorphonuclear leukocytes in the retinal microvasculature have been topographically correlated with regions of capillary occlusion [31]. These alterations are believed to contribute to progressive microangiopathy that includes vascular occlusion and regions of nonperfusion that could make the retina susceptible to hypoxia. It is feasible that the microangiopathy that appears to be partly inflammation dependent is facilitated by the pro-inflammatory isoforms of VEGF. It has been demonstrated that VEGF is chemotactic to monocytes and upregulates ICAM-1 expression, promoting leukostasis [32]. It has been proposed that the pathological neovascularization present in diabetic retinopathy requires the induction of inflammation and leukocyte adhesion to the vessel wall mediated by VEGF-164 isoform [33]. This pro-inflammatory milieu appears to be a prerequisite for induction of the early and potentially progressive pathogenesis of diabetic retinopathy.

Oxidative stress mechanisms and reactive oxygen species have been implicated in the pathophysiology of diabetic retinopathy. The activation of these pathways leads to increased mitochondrial superoxide production in endothelial cells and trigger inflammatory mediators and dysregulated angiogenesis [34]. Poly(ADP-ribose) polymerase (PARP) is involved in oxidative-stress pathways activated during diabetic retinopathy. In diabetic animal models, PARP is linked to hypoxia-induced VEGF overexpression, and PARP inhibitors are able to prevent VEGF overexpression by a posttranslational mechanism [35]. Oxidative stress has been linked to apoptosis of retinal pericytes by the induction of the highly reactive oxoaldehyde, methylglyoxal [36]. Additionally, the pericytes of diabetics demonstrate increased NF-*κ*B, and it is surmised that hyperglycemia activates NF-*κ*B and induces apoptosis of retinal pericytes [37].

Recent evidence have suggested that high glucose modulates TGF-*β* signals in mesenchymal cells linked to Ca(2+)/PKC/MAPKs as well as PI3K/Akt/mTOR signal pathways [38]. The interrelationship between TGF-*β*, pericytes, and the maintenance of a quiescent retinal endothelial cell has previously been evaluated [39]. A subpopulation of pericytes expresses the growth factor TGF-*β*1, and cross-talk signaling with the endothelial cell enhances the expression of VEGFR1 on endothelium imparting a protective effect on the vasculature from oxidative damage [40]. The involvement of mTOR signaling in pericytes could have implications with regards to the angiogenic mechanism(s) that might be involved in pericyte biology and would be of profound relevance during early subclinical stages of diabetic retinopathy.

Loss of pericytes is one of the earliest histopathological lesions as well as a unique feature of diabetic retinopathy [41]. Reactive oxygen species (ROSs) can indirectly activate and promote the nuclear translocation of the pro-inflammatory transcription factor NF-*κ*B via the degradation of the negative regulator IkB-*α* in cytoplasm. The activation of NF-*κ*B leads to translocation into the nucleus where it binds to DNA and modulates the expression of various genes controlling the inflammatory process [42]. Elevated PARP also plays a role in the occurrence of early stage diabetic microangiopathy, such as a cellularity and pericyte degeneration. The proposed mechanism is via the activation of NF-*κ*B and the consequences of initiating downstream effectors such as ICAM-1 which leads to leukostasis [43].

The mTOR inhibitors could exhibit beneficial effects for diabetic retinopathy by suppressing a pro-inflammatory phenotype and modulation of redox sensitive pathways. Suppression of NF-*κ*B by PI3K/Akt-1/mTOR pathway inhibition would have a pronounced regulatory influence on the inflammatory cascade by promoting a generalized anti-inflammatory effect. Some of the mTOR inhibitors, such as rapamycin, have an established immunosuppressive effect. Although this can impart an unfavorable side effect profile, it can be an advantageous attribute if it can be used to suppress the pro-inflammatory phenotype that exists in diabetes. The immunomodulatory attribute of mTOR inhibition could be used to suppress NF-*κ*B expression, which would reduce the expression of downstream pro-inflammatory mediators such as monocyte chemoattractant protein (MCP-1), VEGF, TNF-*α*, IL-1*β*, RAGE, ICAM-1, and vascular cell adhesion molecule (VCAM-1) that are under the regulatory influence of NF-*κ*B. These pro-inflammatory cytokines, chemokines, and adhesion molecules have been demonstrated to play a role in the development and progression of diabetic retinopathy [44]. Suppression of TNF-*α* by omega-3-polyunsaturated fatty acids reduces angiogenesis in a mouse model of oxygen-induced retinopathy as well as implicated in diabetic retinopathy [45]. Thus, NF-*κ*B is a mediator for cytokine-induced inflammatory responses by serving as a central convergent regulator that increases the release of cytokines and other chemotactic factors operant in inflammation.

## 6. Significance of PI3K/Akt/mTOR Inhibition in Proliferative Diabetic Retinopathy

An indication suggesting that the inhibition of PI3K/Akt/mTOR pathway could have beneficial therapeutic effects for the management of proliferative diabetic retinopathy stems from the findings that growth factors known to play major roles in the induction of angiogenesis depend on PI3K/Akt/mTOR for prolonging the cell survival signals that are operant in pathological angiogenesis [46]. The proliferative stage of diabetic retinopathy is ischemia driven in which the hypoxia amplifies the proliferative component of angiogenesis. Signaling via mTOR pathway has been shown to augment mitogen-stimulated vascular cell proliferation and angiogenesis in response to hypoxia [47]. The signaling mediated thru mTOR plays a major role in hypoxia-induced smooth muscle and endothelial cell proliferation. Tissue hypoxia modulates HIF-1*α* hydroxylation and regulates its protein and activity levels [48]. HIF-1*α* induces the expression of various growth factors and genes such as VEGF, VEGF flt-1 receptor, bFGF, PDGF, nitric oxide synthases, angiopoietin 2, and IGF-1 that are established inducers of neovascularization. In ocular tissue, it has been demonstrated that the proangiogenic effects of IGF-1 are mediated via up-regulated VEGF expression obtained by activation of the PI3K/Akt/mTOR pathway and posttranscriptional activation of HIF-*α* [48]. It has been demonstrated that mTOR pathway influences the mechanism on how the same growth factor, such as IGF-1, can exhibit divergent pleiotrophic effects in an HIF-1*α*-dependent manner [49]. For instance, IGF-1 can mediate VEGF expression by mechanisms dependent as well as independent of HIF-1*α*, including stress and cytokine-induced VEGF production [50, 51]. Furthermore, transgenic mice overexpressing IGF-1 in the retina develop vascular alterations that resemble human diabetic retinopathy [52].

Both placenta growth factor (PIGF) and VEGF increase Akt phosphorylation and activate downstream substrates. Experimental blockade of PI3K signal and activation by over expression of adenovirus-mediated phosphatases that disrupt Akt phosphorylation also disrupt angiogenesis. Therefore, several growth factors that have demonstrated a role in the development of the vasculopathy characteristic of human proliferative diabetic retinopathy are linked to the PI3K/Akt/mTOR pathway for the regulation of their expression and activity. The mTOR pathway has also been implicated in other pathobiology of the retina. The dedifferentiation of RPE and subsequent photoreceptor degeneration is associated with mTOR activation. The inhibition of mTOR pathway is able to suppress RPE dedifferentiation as well as preservation of photoreceptor functionality in mice [53].

The recognition that oxygen levels regulate mTOR function and that mTOR is involved in hypoxia-facilitated vasoproliferative responses proposes a relatively novel downstream functional link between hypoxia and mitogenic signaling involved in proliferation of vascular cells [47]. These collective observations suggest that PI3K/Akt/mTOR pathway inhibition would be suited to manage the advanced proliferative stages of diabetic retinopathy where hypoxia-driven vasoproliferative mechanisms predominate in contributing to the vasculopathy.

## 7. PI3K/Akt/mTOR Inhibitors as Potential Therapeutics

**Figure 1:**
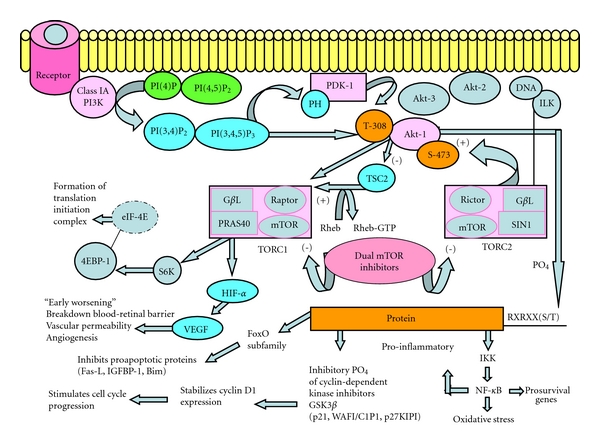
Schematic of the PI3K/Akt/mTOR Pathway. pathway highlights downstream effectors pertinent to the development of diabetic retinopathy along with the benefit of dual inhibitors of mTOR (TORC1 and TORC2) that prevent feedback activation and prevent downstream effectors that are detrimental in the progression of diabetic retinopathy. References consulted for Figure 1: [56], Paloma Pharmaceuticals presentation files, and Angioceutics International.

The inhibition of the PI3K/Akt/mTOR pathway is an attractive therapeutic target for diabetic retinopathy because functionally it is a convergent pathway for a variety of growth factors, pro-inflammatory mediators, and downstream substrates that are regulators of cellular survival processes essential to the initiation and progression of the angiogenic cascade (Figure 1). Novel findings regarding the regulation of VEGF expression in the retina of rodents suggest that hyperglycemia induces VEGF protein expression via eukaryotic initiation factor-4E (eIF4E) and its binding proteins (4E-BP1&2) [54]. Mice null for these proteins did not exhibit increases in VEGF protein initiated by hyperglycemia. The eIF4E and 4E-BP1 proteins are downstream effectors of the regulatory mTOR complex 1 (mTORC1), thereby, implicating a functional role of this pathway in the pathobiology of diabetic retinopathy.

Several inhibitors of the PI3K superfamily have been described [55]. The pharmacologic agents LY294002 and wortmannin both target the p110*α* catalytic subunit of PI3K [56]. Perifosine and PX-866 are lipid-based Akt inhibitors that prevent translocation to the membrane while phosphatidylinositol ether analogs (PIAs) bind to the PH domain of PDK-1. Triciribine (API-2) is selective for Akt-2 inhibition [56]. Targeting proximal pathway components generally result in broad inhibition of downstream signaling cascade and may augment undesirable side effects.

Clinically marketed compounds that modulate a more downstream pathway component are mTOR complex inhibitors and include TORISEL, Afinitor, and Rapamune (rapamycin). The best characterized mTOR complex inhibitor is rapamycin, “a macrolide antifungal compound produced by the soil bacterium *Streptomyces hygroscopicus* isolated from the soil of Rapa Nui (Easter Island)” [55]. Rapamycin interacts with FK506-binding protein and inhibits the activity of TORC1 with extremely high selectivity [55]. Intraperitoneal administration of rapamycin has demonstrated anti-angiogenic efficacy in mice with laser-induced choroidal neovascularization and in oxygen-induced retinopathy [57].

An abbreviated summary of some principal of Akt, and first- and second-generation mTOR inhibitors that have advanced to various stages of clinical development along with selected naturally occurring agents with pending prospects for medical indication are summarized in Table 2.

## 8. Pitfalls, Limitations, and Progress of mTOR Inhibitors

Toxicities associated with various mTOR inhibitors that are particularly pertinent to diabetics include gastrointestinal effects, hematological, decreased glucose tolerance, hyperglycemia, and hypertriglyceridemia. These effects may stem from the involvement of this pathway in the regulation of hexokinase and glycolysis leading to deregulation of glucose and lipid homeostasis [58]. Inroads continue to be made into the mechanistic understanding of some of the more prevalent side effects that have been demonstrated with mTOR inhibitors [59]. The included summary Table 3 highlights many of the reported adverse effects of several mTOR inhibitors from a variety of clinical and preclinical studies [60–65]. The adverse effects are manifested in many organ systems with different incidence rate and duration of drug treatment when administered for systemic exposure. The percent incidence and duration of treatment, when reported as a range in the table, are a compilation from several different studies. Almost all adverse effects are manageable with appropriate clinical intervention or fully reversible upon the discontinuation of the drug. Early reported adverse effects involve cutaneous lesions and oral ulcerations [66–69]. With more prolonged drug use, metabolic [70–72], hematological alterations [73, 74], and renal toxicities [75, 76] can become evident but are generally manageable. Of greatest clinical concern is the development of noninfectious pneumonitis which requires careful monitoring and clinical intervention [77, 78]. One study has reported a high incidence of reversible infertility [79].

The potent anti-angiogenic effects of mTOR inhibitors can have deleterious effects when there is the requirement for physiological processes that are dependent on angiogenesis, such as cutaneous wound healing, menstruation, bone growth, and remodeling of bone following fractures. The inhibition of mTOR pathway could lead to delays in wound healing perhaps linked to modulation of immune responses [80–82]. In murine bone fracture models, Rapamycin has been shown to delay callus formation and reduce biomechanical bone strength during the healing process, but without appreciable detriment to the bone after the period of healing [83]. A special concern arises in the treatment of young children because experimental studies have shown that rapamycin can inhibit vascularization at the epiphyseal plate of long bones resulting in stunted growth in rats [84]. However, it is rare for this age group to develop diabetic retinopathy and therefore not a likely patient population that would be of concern for this mode of therapy. 

Many potential side effects can be avoided by temporary cessation of drug administration during periods for which the patient has special transient considerations. Careful monitoring must be given when treating patients in the acute phase of wound healing, in diabetics with a heightened risk for the development of foot ulcers, and those with bone fractures. Based on our current understanding of the mTOR pathway's role in wound healing, it would appear prudent that early and close monitoring and perhaps even transient discontinuation of drug treatment is warranted in cases where patients are experiencing an active resolution of a cutaneous wound or other physiological healing processes that are angiogenic dependent. The implementation of careful patient counseling and adaptive drug regiment plan should be effective in minimizing or preventing this manageable side effect component of mTOR inhibitors. 

As we acquire a greater understanding of the mechanistic basis for the associated side effects with this class of drugs, it will expand the therapeutic utility and diversify the potential medical applications such as for the management of diabetic retinopathy.

## 9. Therapeutic Potential of Second-Generation mTOR Inhibitors

The PI3K/Akt/mTOR pathway has proximal and distal feedback signaling and although mTOR is downstream effector of Akt, the mTOR complex 2 (TORC2) can phosphorylate Akt which then activate Akt via a feedback mechanism [85]. Rapamycin and early spin-off analogs (rapalogs) mTOR inhibitors had the limitation that they did not affect mTORC2; consequently, duration of inhibition was shortened due to feedback activation of Akt, The mechanism by which “rapalogs” selectively inhibit mTOR complex 1 (TORC1) has been elucidated in detail and involves mTORC1-dependent phosphorylation of 4E-BP1 and S6K1 through distinct mechanisms [86]. Rapamycin, perhaps as a consequence of feedback activation of Akt via TORC2, has exhibited a paradoxical increase in VEGF and Flt-1 protein levels in response to pathway inhibition. This feature would appear to be problematic for the long-term management of diabetic retinopathy. This feedback loop diminishes the extent of pathway blockade and has resulted in limited efficacy of these therapeutic agents in the past. However, newer generation mTOR inhibitors do not present this potentially detrimental feedback issue.

A successful approach to drug design that circumvents the limitations of previous mTOR inhibitors due to feedback activation of Akt has been developed. Selective and potent novel inhibitors of mTOR which exhibit dual inhibition of mTORC1 as well as mTORC2 have demonstrated high efficacy in preventing feedback-loop activation of the pathway and rendered improvements in outcome measures. The sophistication of the armamentarium of drugs now available include highly specific mTOR inhibitors, dual PI3K/mTOR inhibitors [87], as well as AKT inhibitors that may possess ATP-competitive or ATP-independent allosteric modulators [88]. 

Technological breakthroughs in drug design continue to improve the approach to target both PI3K and mTOR pathways via hybrid inhibitors such as diester-linked conjugates capable of bridging two inhibitors in combination, with the potential to enhance efficacy [89]. Dramatic improvements in mTOR-targeting specificity and selectivity continue to be achieved by molecular modeling and synthetic chemical methods [90]. 

Although an extensive inclusion of the various types of mTOR inhibitors is beyond the scope and main focus of this review, there are numerous excellent review articles available. The interested reader is referred to those articles for further information regarding general overviews of mTOR inhibitors [91–93], emphasis on development of dual mTOR inhibitors [94–96], functional consequences of mTOR inhibition [97], mTOR inhibitors in clinical development [98, 99], and discussion of some natural mTOR inhibitors [100]. Green Tea [101] and epigallocatechin gallate (EGCG) [102], both natural mTOR inhibitors, have been shown to impart protective effects in diabetic retinopathy. However, the benefit that is derived from green tea and EGCG appears to be predominantly mediated by their potent antioxidative properties. The polyphenol resveratrol also has mTOR-modulating properties and has exhibited cytoprotective effects and inhibition of VEGF secretion in human retinal ARPE-19 cells [103]. The benefit to diabetic retinopathy stemming from these compounds that may be attributable to the ancillary effect of inhibition of the mTOR pathway has not been documented and remains to be elucidated. 

Of the two mTOR inhibitors in NIH clinical trials for ocular indications (Tables 1 and 2) neither is targeting diabetic retinopathy per se as an indication although preclinical data strongly suggest that they possess varied pharmacological features that would make them efficacious candidates for treatment of diabetic retinopathy. One of these inhibitors, Sirolimus (Perceiva), has recently completed (January 29, 2011) a fast-track designated NIH sponsored pilot study with five participants to evaluate treatment option for diabetic macular edema. The primary outcome measure is changed in visual acuity at six months relative to baseline. Final data collection has been completed, and results are eminently pending. Separate Phase 2 studies evaluating Perceiva for neovascular AMD and dry eye syndrome are also pending. Limitations that may confront Perceiva as a clinical agent are the reported immunosuppressive effects and that the anti-angiogenic effects are predominantly cytostatic rather than anti-angiogenic or angiolytic. 

The other inhibitor, Palomid 529, a small molecule synthetic non-steroidal compound with a chemical structure derived from dibenzo[c]-chromen-6-one, is a first-in-class allosteric dual mTORC1 and mTORC2-*dissociative* inhibitor that abrogates compensatory feedback loop activation. The mechanism of action is unique in that it dissociates the various proteins in the mTORC1/C2 complex rather than inhibiting via catalytic competitive inhibition. This presumably imparts broader inhibitor activity. Palomid 529 has had extensive characterization of preclinical pharmacokinetic, biodistribution, and efficacy testing involving ocular studies. Muller cell proliferation and glial scar formation is reduced following experimental retinal detachment in a rabbit model using Palomid 529 [104]. The safety profile for Palomid 529 is excellent without apparent adverse effects. Concentrations of the drug remain detectable in the retina and choroid for at least six months after last dosing. Therefore, the frequency for repeat subconjunctival or intravitreal administration is minimized along with the risk of iatrogenic ocular complications. 

**Figure 2:**
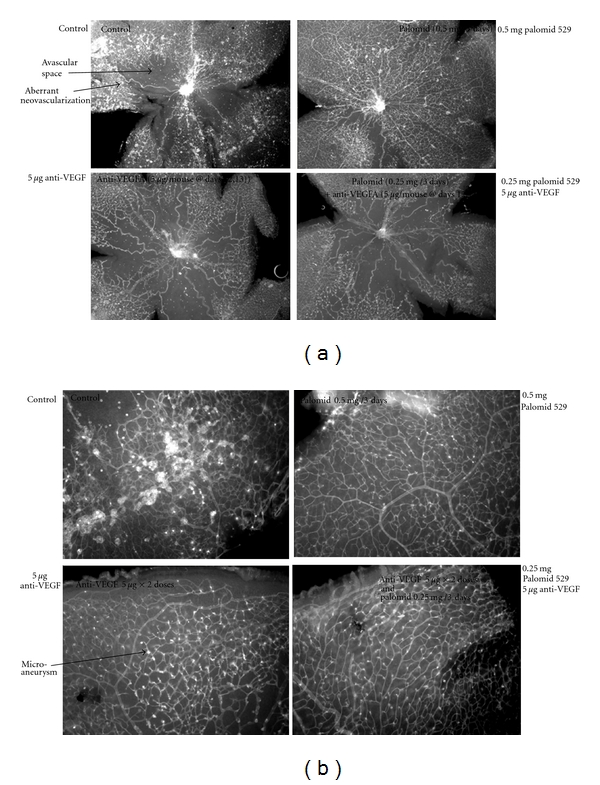
Oxygen-induced retinopathy model-retinal flat mount. Seven-day old SV129 mouse pups were put at 70% oxygen for 5 days, removed to normal air, and injected intraperitoneal with Palomid 529 for 5 days. The animals were then sacrificed and the eyes removed and fixed in 10% formalin for 30 min. The retinas were isolated, blocked for 1 hr in PBS containing 1% BSA, 1% goat serum, and 0.5% TX-100, stained overnight with 10 *μ*g/mL BS-1 lectin labeled with FITC (Sigma-Aldrich), washed in PBS and flat mounted.

Clinically relevant adverse events have been experienced with the use of TORC1 inhibitors, Sirolimus, and its analogs, when administered via systemic administration as described in Table 3. However, as retinal therapeutic agents are routinely administered via a targeted approach, that is, intravitreal or subconjunctival, many of these issues would not be encountered since the local dose of drug administered would not reach sufficient levels in the systemic circulation to cause toxicities. With Palomid 529, such toxicities have not been observed to date in its ongoing human Phase I age-related macular degeneration study where administration was either intravitreal or subconjunctival (Paloma Pharmaceuticals, personal communication). Dual mTORC1/mTORC2 inhibitors might be expected to effectively induce complete blockade of the PI3K/Akt/mTOR pathway, a signaling cascade found in all cells necessary for normal homoeostasis, thereby exerting toxic effects. Relative to Palomid 529, no toxicity was noted in non-GLP or GLP toxicology studies in dogs and rats when the drug was administered intravenously at dose levels well above that which had been shown to exert activity in a variety of animal models of ophthalmic or oncologic disease [105]. No dose-limiting toxicities were found when Palomid 529 was administered in a dose-ranging intravitreal non-GLP or GLP studies in dogs and rabbits (Paloma Pharmaceuticals, personal communication). Relative to Palomid 529, it is possible that its inhibitory effects on the PI3K/Akt/mTOR pathway are not to induce an absolute blockade of the pathway, but to reduce its pathological up-regulation to a normal level. In the oxygen-induced retinopathy model (retinopathy of prematurity model), an established surrogate animal model for evaluating hypoxia-induced progressive vasculopathy reminiscent of mechanisms operant in diabetic retinopathy, Palomid 529 inhibited pathological neovascularization, see Figure 2(a).

In this model, when Palomid 529 is compared head to head with a murine anti-VEGF antibody, the anti-VEGF antibody treatment appears to inhibit both pathological and normal angiogenesis while Palomid 529 inhibits predominantly pathological angiogenesis. This is shown by presence of avascular space around optic nerve in control, increased with anti-VEGF treatment but essentially lacking with Palomid 529 treatment. This observation suggests that the inhibitory actions of Palomid 529 influencing the PI3K/Akt/mTOR pathway is mediated by normalizing the signaling activity level of this pathway rather than promoting a suppressive blockage leading to subnormal function. In support of this viewpoint is the observation that neonatal vascularization in the oxygen-induced retinopathy mouse pups was not adversely affected and perhaps eases concerns regarding the induction of adverse events in young patients when using Palomid 529.

In addition, upon closer inspection at higher magnification, anti-VEGF antibody did not appreciably inhibit glomeruloid formation (“microaneurysms”), while Palomid 529 showed significant inhibition of this vascular malformation, see Figure 2(b). 

Palomid 529 has completed 4 of 6 cohorts of the company's ongoing intravitreal Phase 1 human age-related macular degeneration trial. The NEI is also conducting its own Phase I trial in age-related macular degeneration with subconjunctival administration. Preliminary results in the intravitreal study have shown significant reduction of retinal thickness as evidenced by OCT in two of the three patients at the 4th cohort (0.5 micrograms). Positive data has also been observed with the NEI trial. The outcome of these trials will be very instructive with regards to future application of this drug, other drugs of its class, and to other angiogenic ocular diseases. 

Clinical trial data on safety and efficacy of dual mTOR inhibitors is emerging, particularly for the treatment of a variety of cancers. There have been widespread concerns that the novel dual mTOR inhibitors with their potent capacity to cause extensive and diffuse blockade of downstream signaling will exhibit additional and perhaps unpredictable side effects beyond what has already become apparent from the side effect profile of the early generation mTOR inhibitors. However, from the limited clinical data that has emerged using dual mTOR inhibitors, the prognostic outlook for the utility of these agents in providing improved therapeutic outcomes with reduced tachyphylaxis appears encouraging [106]. For the treatment of leukemia, the dual mTOR inhibitor NVP-BEZ235 has exhibited the potential to act synergistically to augment the effect of other chemotherapeutic agents [107] and appears to facilitate bone mineral-matrix deposition thereby countering the potential for bone loss with certain tumors [108]. In gliomas, this dual mTOR inhibitor has not demonstrated toxicities and exhibits potent anti-angiogenic effects [109].

## 10. What Future Frontiers and Direction are Available for mTOR Inhibitors with the Aim to Treat Diabetic Retinopathy?

It has been suggested that mTOR inhibition in the setting of hyperinsulinemia and type 2 diabetes would be a particularly attractive therapeutic modality [110]. The use of mTOR inhibitors in diabetics is suggested despite this class of drugs inducing alterations in glucose and lipid metabolism, which can be offset and carefully monitored and corrected with concomitant glucose-lowering and/or lipid-lowering pharmacological agents that have good efficacy and low toxicity.

From a drug development standpoint, the PI3K/Akt/mTOR pathway has presented some unique challenges [111]. The high degree of evolutionary conservation of the PI3K/Akt/mTOR pathway across species is a reminder that it subserves a myriad of critical and essential biological functions, and as such it must be targeted with high specificity in the aim of decreasing toxicity. However, the pathway has extensive interactions with other biological pathways and is subject to a rather complex self-regulating negative feedback loop [111]. The existence of multiple and oppositional regulators contributes to the complexity on how best to achieve an efficacious inhibition of pathway signaling. For instance, rapamycin has exhibited limited efficacy as a consequence of negative feedback activation of PI3K/Akt in ocular applications aimed at modulating cellular proliferation in uveal melanoma [112]. This finding underscores the future need for molecules that exhibit dual inhibition of mTORC1/C2 complexes to circumvent limitations imparted by feedback regulation.

In order to prevent or delay drug resistance and minimize ancillary side effects of mTOR inhibition, selective dual inhibitors of mTOR complexes as well as combination therapy with other agents such as VEGF antagonists will be crucial for the development of new therapeutic options to manage the complex vasculopathy of diabetic retinopathy. A significant therapeutic opportunity exists in that mTOR inhibitors reduce VEGF mRNA stability [113], thereby, providing a rational basis to explore whether combination therapy of mTOR inhibitors and anti-VEGF agents can produce additive or synergistic beneficial effects in regulating the angiogenic component of diabetic retinopathy. Combination of mTOR inhibition with VEGF antagonism has demonstrated an augmented effect in suppressing endothelial cell growth in prostate tumor cells [114] and angiogenesis in a model of oxygen-induced retinopathy. 

Dual mTOR inhibitors capable of synergizing with anti-VEGF therapeutics that either inhibit a distinct regulatory site on the same pathway or inhibit a parallel prosurvival pathway would provide a broader mechanistic intervention of the angiogenic process. Because mTOR inhibitors have a direct anti-angiogenic effect, mediated via modulation of HIF-1*α*, it may be possible to approach anti-angiogenic therapy from a dual approach in combination with anti-VEGF monoclonal antibodies (bevacizumab, ranibizumab, and pegaptanib) or VEGF-trap while minimizing the potential for overlapping toxicities and at the same time selectively targeting the operant mechanism(s) in the pathobiology of diabetic retinopathy. 

Several Phase I studies have investigated the safety profile of combination therapy using bevacizumab and mTOR inhibitors sirolimus, everolimus, or the dual mTOR inhibitor WYE-125132 in cancer patients [115–117]. Preliminary data suggest that combination therapy of these agents is a feasible therapeutic modality with tolerable side effects. In general, the prevalence and severity of observed toxicities with combination of these drugs were no greater than what has been observed and associated with each individual drug. Of therapeutic benefit was the potential to lower the dose of each individual agent to improve dose-limiting toxicities over the long run while retaining or even enhancing efficacy of treatment. Future trials will need to elucidate whether combination therapy versus serial drug treatment regiment can also offer an alternative attractive treatment option for disease management. 

An analogous approach can be taken by linking mTOR inhibitors with other antagonists or agents where the mechanism of action targets an alternate pathway, thereby augmenting the potential for additive or synergistic outcomes on efficacy measures. The combinatorial drug approach with mTOR inhibitors can be extended to be coadministered with an entire class of anti-inflammatory agents as combination therapy. The mTOR inhibitors in combination with Nepafenac, currently in clinical trials for non-proliferative diabetic retinopathy and macular edema, would appear to be a feasible combinatorial-drug approach to combat diabetic retinopathy. Experimental findings using topical 0.3% Nepafenac 4x/day in diabetic rats for up to 9 months has demonstrated reductions in superoxide, cyclooxygenase-2, PGE-2, and leukostasis and prevention of functional changes in oscillatory potential as well as vasculopathy including apoptosis, regions of acellularity, and degeneration of pericytes [118].

The multi-drug approach might provide the therapeutic advantage that lower doses of each of the combined agents would be required for efficacy with the benefit of minimizing potential toxicities. This strategy can be justified on the evidence that extensive cross-talk of pathways underlie the angiogenic signaling cascade and that the vasculopathy innate to diabetic retinopathy involves a myriad of initiators. Particularly, attractive would be the combinations of mTOR inhibitors with triamcinalone (Kenacort) or dexamethasone (Ozurdex) both of which have developed either scleral or intravitreal sustained drug delivery formulation and first-in class biodegradable device technologies for drug delivery to the retina. 

Several studies have investigated the benefit of combining mTOR inhibitors with established glucocorticoid anti-inflammatory agents in cancer patients. The mTOR inhibitors not only potentiate the apoptotic effect of steroids, but confer enhanced sensitivity to glucocorticoids, thereby, potentially allowing sustained efficacious and chronic use of these drugs in ophthalmology to treat ocular angiogenic and inflammatory diseases without having to increase dosage over time. The clinical utility of glucocorticoids in ophthalmology is extensive but is hampered by side effects as well as the development of glucocorticoid resistance imposing a limit on the duration of use and clinical utility. 

The combined use of rapamycin with dexamethasone appears to impart the benefit of not developing resistance to the biological effects of dexamethasone as well as enhancing the proapoptotic caspase-3 signaling [119]. The molecular pathway by which mTOR inhibitors are able to augment the pro-apoptotic effects of glucocorticoids and confer enhanced sensitivity to dexamethasone in a variety of cell lines has recently been elucidated. Rapamycin promotes the dissociation of the Bim-Mcl-1 complex to promote dexamethasone-induced apoptosis [120] and by antagonizing the effect of glucocorticoids on the phosphorylation state of 4E-BP1 at Ser65 and p27 upregulation [121]. The mTOR inhibitor CCI-779 in combination with dexamethasone also augments the apoptotic effect of the anti-inflammatory agent [122]. The combination of mTOR inhibitors with COX2 inhibitors promotes a synergistic effect in suppressing tumor angiogenesis that allows subtoxic doses of each agent while retaining efficacy in the clinical management of the disease [123]. 

Transscleral delivery of triamcinalone and Lucentis has been successfully applied in animal models using electrically facilitated macroesis methodology [124]. Dexamethasone has been shown to suppress the release of various pro-inflammatory and pro-angiogenic cytokines from retinal pericytes [125]. Given the prominent role that pericytes play in the etiology of diabetic retinopathy, this could be a significant novel therapeutic avenue to address the early pathological changes and influence disease sequelae. Implants with sustained release of anti-inflammatory agents have been successfully applied when placed in the suprachoroidal space to treat uveitis [126]. Biodegradable hydrogels for implantation in a subconjunctival location have the potential for chronic periocular delivery of drugs to treat diabetic retinopathy [127].

## 11. Multiple Options and Opportunities to Minimize Undesirable Systemic Side Effects

Due to anatomical and physiological barriers, the eye presents a myriad of challenges as a target organ for drug delivery. Recent advances in drug delivery technology including formulation, polymer chemistry, nanotechnology [128], microdrug devices [129], and surgical advancements have permitted the exploration of several unique options and opportunities for topical ocular drug administration. These approaches expand the usefulness of many drugs to treat ocular diseases which otherwise would fail to demonstrate efficacy or would exhibit substantial systemic adverse effects that would preclude their clinical use. Significant advances in drug delivery methodology have improved drug retention time, bioavailability, and enhanced trans-scleral or corneal penetration. These technologies include the use of hydrogels [130], mucoadhesive polymers [131], cyclodextrins, nanocomposite formulations [132], micellar and lipid nanoparticles [133], niosomes [134], microemulsion, microspheres, and prodrug derivatization [135]. The reader is referred to the cited references for a comprehensive coverage on the topic of ophthalmic drug delivery and the highlighted techniques currently available. 

The optimal drug delivery approach depends, to a substantial extent, on the physiochemical and pharmacokinetic properties of the pharmacological agent to be administered. Some of the highlighted techniques, although optimized for ocular surface or anterior pole diseases, have resulted in sufficient enhancement of drug penetration that they also have utility for pharmacological treatment of ocular diseases of the posterior segment. Several of the anti-inflammatory and anti-VEGF pharmacological agents that are proposed in this review to be used in combination with mTOR inhibitors have been administered to the ocular surface using one of the described drug delivery or formulation technologies to treat retinal diseases. For instance, nanocomposites have been used to deliver Diclofenac [136], and topical administration of Nepafenac has been shown to reduce the extent of microangiopathy in animal models of diabetic retinopathy [118] and oxygen-induced retinopathy [137]. Nanoparticle technology has been employed to enhance the surface penetration of hydrophobic compounds such as glucocorticoids to posterior ocular structures [138]. Furthermore, nanoparticles injected into the vitreous have demonstrated intraretinal localization for several months after initial dosing, thereby, serving as a localized drug release depot [139]. 

A microparticle formulation containing an antagonist to a leukocyte antigen applied topically to the ocular surface has demonstrated sufficient ocular penetration to influence leukocyte dynamics and vascular leakage in the retina, both manifestations of diabetic retinopathy [140]. Use of electrical currents applied to the ocular surface in the technique of iontophoresis or macroesis are being used experimentally to successfully obtain retinal concentrations of triamcinalone and ranibizumab when applied on the sclera [141]. 

Additional techniques and methods have been optimized with the specific aim of treating diseases of the posterior pole [142–145]. These approaches permit a sustained and stable multifold increase in drug concentration to reach the retina without inducing systemic side effects while improving therapeutic outcome. Sustained-drug release intraocular implants for delivery of triamcinalone [146] and polylactic-glycolic acid microspheres to deliver dexamethasone [147] to treat diabetic retinal complications and inflammation have been used successfully [148]. Lipid nanoparticles have been used to deliver bevacizumab directly into the vitreous of rabbits with the result of chronically increasing the concentration and bioavailability of the drug in the vitreous several folds [149].

These biodegradable or nonbiodegradable intraocular implants can be placed in the vitreous or via cannulation in the suprachoroidal space [150] to lower the frequency of intraocular injections, improve drug bioavailability in the retina, and circumvent the potential for systemic side effects. 

Of particular interest, in light of the theme of this review, is the use of microemulsion to enhance the corneal permeation of the mTOR inhibitor everolimus with sustained stability of the drug [151] and the use of thermoresponsive hydrogels that have been used to deliver bevacizumab and ranibizumab [152]. 

While it is unlikely that a single drug will be efficacious for managing all the various stages of diabetic retinopathy, combination or sequential therapeutic agents are more apt to yield beneficial results. Combinatorial use of a dual mTOR inhibitor with anti-VEGF antibodies or VEGF-trap could neutralize cross-talk inducers of VEGF expression and be a powerful combination approach to ocular anti-angiogenic therapy. Compelling evidence for enhanced efficacy of combined drug therapy to combat ocular angiogenesis has been previously presented, and the evidence underscores the extensive overlap of regulatory signaling involved in the angiogenic cascade [153]. Potent synergistic effects of combining angiostatic molecules aimed at divergent aspects of the angiogenic process have resulted in more extensive suppression of the vasculature without adverse effects on established quiescent vasculature [154].

The combination of mTOR inhibitors with anti-inflammatory agents also provides a rational-based approach to combat ocular angiogenesis and early hemodynamic changes in the retina. The mTOR inhibitors are uniquely suited to address both early and advanced manifestations of diabetic retinopathy. The mTOR inhibitors have the potential to delay or prevent the progression of retinal microangiopathies by helping to avert breakdown of blood-retinal barrier by modulating HIF-*α*-mediated downstream activation of growth factors. As the disease progresses and the characteristic lesions are proliferative in nature, the inhibition of PI3K/Akt/mTOR pathway would provide an effective means to abrogate neovascularization by shutting down prosurvival growth factors, modulating the inflammatory cascade, preventing angiogenesis, and promoting apoptosis of nascent vessels. 

As we continue to unravel the complexity of the initiating factors that contribute to the microangiopathy observed in progressive diabetic retinopathy and gain further understanding of the natural progression of the disease it is imperative that emerging therapeutics like mTOR inhibitors be well contemplated in the context of their mechanism of action, stage progression of the retinopathy, and the critical timing of pharmacological intervention. A drug can be ineffective or even result in adverse effects if implemented during an inappropriate stage of disease progression. Therefore, managing of the complex vasculopathy in diabetic retinopathy will require elucidating the proper timing of when to administer the therapeutic agent for optimal efficacy. Regardless of the enigmatic components that remain with regards to the elucidation of the molecular pathways operant in diabetic retinopathy, these novel classes of therapeutics are likely to produce better patient outcome for managing the widespread and devastating disease of diabetic retinopathy. The mTOR inhibitors, particularly when combined with other pharmacological agents would appear to be a promising therapeutic modality.

The second-generation mTOR inhibitors discussed in this review are well positioned to fulfill several key criteria for being an optimal therapeutic for treatment of ocular angiogenesis: (1) targets neovascularization by specific mechanism, (2) delays or prevents the angiogenic phase of the disease, (3) demonstrate specificity and selectivity for aberrant vessels, (4) has a formulation for long-term delivery with no apparent toxicity associated with chronic administration, (5) stabilize, or prevent further deterioration of vision, (6) prevent or delaying late-stage complications of the disease such as detachment and scarring.

##  Disclosure

Dr. Jacot has no proprietary or commercial interest in any materials presented in this review. Dr. Sherris is President and Chief Executive Officer of Paloma Pharmaceuticals, Inc. and has proprietary and commercial interests in Palomid 529 mentioned in this review.

## Figures and Tables

**Table 1:** Number of open NIH trials by indication.

	Non-proliferative phase of diabetic retinopathy	Proliferative phase of diabetic retinopathy	Diabetic macular edema	Other*
Anti-VEGF agents				
Avastin	2	6	12	VH (1)
Lucentis	2	7	16	ARMD (3); RVO (1); rubeosis (1)
Macugen		1	3	Uveitis (1)
Anti-inflammatory agents				
Bromfenac			1	
Dexamethasone	1		1	
Diclofenac			1	
Ketorolac		1	2	
Minocycline			1	
Nepafenac	1		2	
Triamcinalone	1		7	
mTOR inhibitors				
Palomid 529				ARMD (2)
Sirolimus			1	ARMD (1); uveitis (1)
Laser therapy				
Micropulse			2	
Pan laser	4	8	4	
PASCAL laser	4	4	2	

*VH: vitreous hemorrhage, ARMD: age-related macular degeneration, RVO: retinal vein occlusion, Note: a particular trial can have more than one indication or therapeutic agent under investigation.

**Table 2:** Principal PI3K/Akt/mTOR Inhibitors in clinical development.

Name and general classification	Manufacturer or source	Clinical stage	Indication
*Akt inhibitors*			
SF1126 (RGD peptide + LY294002 a morpholino derivative of quercetin)	Semaphore Pharmaceuticals	Phase 1	Chronic lymphocytic leukemia
Perifosine (KRX-0401)	Keryx Biopharmaceuticals, Inc	Phase 2 and Phase 3	Colorectal cancers; multiple myeloma; other multiple cancer types
PX-866	Oncothyreon	Phase 1 & 2	Solid tumor disease; Glioblastoma
Triciribine (API-2; TCN-PM; VD-0002)	VioQuest Pharmaceuticals	Phase 1	p-AKT-positive solid malignancies
*Rapamycin (and analogs)*			
Everolimus (RAD001; Zortress; Afinitor)	Novartis Pharma AG	Marketed 2009	Advanced renal cell carcinoma after failure with sunitinib or sorafenib; subependymal giant cell astrocytoma (SEGA)
Deforolimus (AP23573; MK-8669; Ridaforolimus)	ARIAD Pharmaceuticals; Merck	Phase 3 (SUCCEED TRIAL)	Metastatic soft-tissue and bone sarcomas
Sirolimus (Rapamycin; MSR001; Rapamune; Perceiva)	Santen Inc; MacuSight, Inc.Wyeth Pharmaceuticals	November 2010	Prophylaxis of organ rejection in patients >13 years old receiving kidney transplant
Temsirolimus (CCI-779; TORISEL)	Wyeth Pharmaceuticals	Marketed 2007	Advanced renal cell carcinoma
*Dual mTOR inhibitors*			
AZD8055	Astra Zeneca	Multiple Phase 1 and multiple Phase 2	Advanced hepatocellular carcinoma; advanced solid malignancies and lymphomas
NVP-BEZ235	Novartis Pharma AG		Pancreatic cancer
WYE-125132	Wyeth Pharmaceuticals	Multiple Phase 1 and Phase 2	Advanced malignancies
Palomid 529	Paloma Pharmaceuticals Inc.	Phase 1	Age related macular degeneration (intravitreal and conjunctival administration)
PKI-179	Pfizer; Wyeth	Phase 1	Advanced malignant solid tumors
PKI-402	Pfizer		
PKI-587 (PF-05212384)	Pfizer	Phase 1	Incurable cancer
*Natural mTOR inhibitors*			
Epigallocatechin gallate (EGCG) (flavonoid polyphenol)	Green tea	Pre-clinical	Pending
Caffeine	Multiple and varied	Pre-clinical	Pending
Celastrol	Thunder of God vine (Tripterygium wilfordii)	Pre-clinical	Pending
Curcumin (diferuloylmethane)	Indian spice turmeric	Pre-clinical	Colon cancer (?)
Hispidulin (flavonoid molecule)	Artemisia vestita Saussurea involucrate	Pre-clinical	Pending
Resveratrol	Skin of red grapes	Pre-clinical	Pending

*Note references used to compile table include: Drugs.com, Searchmedica.com, Clinicaltrials.gov, PubMed.gov, and FDA product information inserts when available.

**Table 3:** Reported adverse effects of mTOR inhibitors.

General disorder classification	Specific adverse event	Reported percent incidence	Duration on Meds (days)	Agent	Clinical management options	Outcome
Cutaneous	Delayed wound healing			Multiple mTOR inhibitors		Manageable
	Skin rash			Ridaforolimus		Manageable
	Spongiotic dermatitis with eosinophils		14	Temsirolimus	Clobetasol	Resolution
Hematological	Thrombocytopenia	8–22.7		Everolimus and sirolimus	Interleukin 11	Manageable
	Anemia (microcytic)	9–20		Temsirolimus and everolimus and sirolimus	Erythropoietin	Manageable
	Leukopenia	18.2		Everolimus and sirolimus	Colony stimulating factor	Manageable
Hepatic	Transaminase Elevations	10		Everolimus	Periodic liver function tests	
	Alkaline phosphatase elevations	8		Everolimus	Periodic liver function tests	
Immunological	Severe infections	2.3				
Metabolic	Hypercholesterolemia	50		Sirolimus and everolimus	Statin therapy	Manageable
	Hypertriglyceridemia	31.8		Sirolimus and everolimus	Fibrates	Manageable
	Hyperglycemia	11		temsirolimus	Glucose lowering agents	Manageable
	Fatigue	11		Temsirolimus		
	Hypophosphatemia	5		Temsirolimus		
	Hyperlipidemia	5		Everolimus	Lipid lovering drugs	
Oral	Ulcerations	66	5	Deforolimus	Discontinuation	Reversible
	Mucositis	6			Discontinuation	Reversible
	Aphthous stomatitis	6			Discontinuation	Reversible
Pulmonary	Noninfectious pneumonitis	4.8–18	60–1500	Sirolimus and everolimus	Withdrawal, antibiotics and steroids	Reversible
	Dyspnea	9				
	Grade 1 (asymptomatic)	3.3		Everolimus	Reduction	Reversible
	Grade 2 (Ok daily living)	6.6		Everolimus	Withdrawal	Reversible
	Grade 3 ( oxygen indicated)	3.6		Everolimus	Withdrawal, antibiotics and steroids	Reversible
	Grade 4 (life threatening)	0		Everolimus	None	
Renal	Proteinuria	1.6–4.1	540–1500	Sirolimus and everolimus	Withdrawal	Reversible
	Proteinuria (grade 3 to 4)	25	273	Bevacizumab + everolimus	Discontinuation	Reversible
Reproductive	Infertility (oligospermia)	66.6	150–360	Sirolimus	Discontinuation	Full recovery
	amenorrhea	14.2	150–360	Sirolimus	Discontinuation	Reversible
